# Trainability of Health-Related and Motor Performance Fitness in Adults with Cystic Fibrosis within a 12-Month Partially Supervised Exercise Program

**DOI:** 10.1155/2021/5581812

**Published:** 2021-03-09

**Authors:** Matthias Welsner, Wolfgang Gruber, Uwe Mellies, Margarete Olivier, Sivagurunathan Sutharsan, Christian Taube, Stefanie Dillenhoefer, Cordula Koerner-Rettberg, Florian Stehling

**Affiliations:** ^1^Department of Pulmonary Medicine, University Hospital Essen-Ruhrlandklinik, Adult Cystic Fibrosis Center, University of Duisburg-Essen, Essen, Germany; ^2^Pediatric Pulmonology and Sleep Medicine, Cystic Fibrosis Center, Children's Hospital, University of Duisburg-Essen, Essen, Germany; ^3^Department of Pediatric Pneumology, University Children's Hospital, Ruhr University, Bochum, Germany

## Abstract

**Background:**

Regular physical activity plays an important role in the treatment of patients with cystic fibrosis (CF). This study is aimed at investigating the effects of a 12-month partially supervised exercise program on attributes of health-related and motor performance fitness, lung function (ppFEV1), BMI, and habitual physical activity (HPA, steps/day) in adults with CF.

**Methods:**

Attributes of health-related and motor performance fitness were examined at the beginning (T0), after 6 (T1), and 12 months (T2) on the basis of five test items: forward bend (FB), bent knee hip extension (HE), plank leg raise (PLR), standing long jump (SLJ), and standing on one leg (OLS). Additionally, we recorded HPA by accelerometry, peak exercise performance (*W*_peak_) by an incremental cycle test, ppFEV1, and BMI. During the first six months, there was close supervision by an experienced sport therapist.

**Results:**

26 CF patients (8 female, mean age 26.5 ± 7.9 years; ppFEV1 53.7 ± 21.0) completed the exercise program. Significant improvements were recorded from T0 to T1 (FB: *p* ≤ 0.05; PLR, OLS: *p* ≤ 0.01) and from T0 to T2 (FB, PLR: *p* ≤ 0.01 and HE, OLS: *p* ≤ 0.05). *W*_peak_, ppFEV1, BMI, and HPA showed no significant improvement between the single test points and over the entire study period (all *p* > 0.05).

**Conclusion:**

Our results show trainability of adults with CF in aspects of health-related and motor performance fitness during a partially supervised exercise program. Close supervision positively influences the results. Using a simple test setup seems to be a promising tool for evaluating the effects of exercise programs in CF and could serve as an additional outcome parameter in future clinical trials. Trial registration: ClinicalTrials.gov (retrospectively registered May 8, 2018).

## 1. Background

Cystic fibrosis (CF) is an autosomal recessive multiorgan disease caused by mutations in the cystic fibrosis transmembrane conductance regulator (CFTR) gene, occurring in nearly 1/3500 births [1]. The encoded protein is an anion transporter located on epithelial surfaces. An absence or a reduction in quantity leads to a disturbed transport of chloride and bicarbonate ions which is resulting in abnormal viscous mucus and therefore multiorgan dysfunction [2]. Morbidity and mortality are mainly influenced by the pulmonary involvement [3]. Due to advances in care, life expectancy of patients with CF has increased significantly over the last decades [4].

Physical activity (PA) and exercise training are substantial components in the therapy of patients with CF [5] as they are able to slow down disease progression as well as improve lung function, bone mineral density, weight, and health-related quality of life (HRQoL) [6]. In this context, PA is described as “any bodily movement produced by contraction of skeletal muscle that substantially increases energy expenditure” [7]. PA can be performed during leisure time according to personal interests, needs, and offers [8]. An appropriate dose-response relationship, e.g., heart rate (HR) > 70% of maximum HR, is recommended for patients with CF to improve physical performance [9].

In contrast to PA, physical fitness (PF) is a set of attributes that people possess or achieve [8]. PF splits up in health-related fitness and motor performance fitness [7, 10, 11]. Health-related fitness includes cardiorespiratory endurance, muscular strength, flexibility, and body composition, whereas balance, coordination, speed, agility, and power are attributes of motor performance fitness [12]. Partially supervised exercise programs can enhance PA and PF [5, 9] and showed long-term positive effects in children and adults with CF even after the end of the supervision [13].

Moreover, a higher activity level in everyday life (habitual physical activity, HPA) and improved aerobic performance as an attribute of health-related fitness are associated with a higher probability of survival [14, 15].

The effects of exercise programs focusing on strength and endurance as part of health-related fitness are often assessed by maximum oxygen (VO2_max_) or peak oxygen (VO2_peak_) uptake defined by cardiopulmonary exercise testing (CPET) [16, 17]. VO2_max_ and VO2_peak_ are direct measurements to assess cardiorespiratory endurance and strength of lower limbs. Those parameters have been shown to predict CF-related mortality [14, 17, 18]. Furthermore, CPET is regarded as the gold standard for measuring physical performance in CF [19]. Alternatively, if not feasible, an incremental cycle ergometer test without gas exchange with determination of peak exercise capacity (*W*_peak_) and O2 saturation is suggested as “second-gold” standard to determine maximum exercise capacity [19].

However, little is known about the effects of an exercise program regarding different aspects of motor performance fitness. In this respect, children and adolescents show trainability with regard to motor performance fitness as recently published by our group [20].

In adults, the Senior Fitness Test is an easy to carry out functional fitness test without expensive equipment [21]. It is widely used to determine aspects of motor performance fitness in older adults. However, the Senior Fitness Test is therefore less suitable for younger people and adolescents as well as adults with CF with often nearly normal or normal PF. In children, the Deutsche Motorik Test (DMT) can be used as a tool to assess PF, but reference values only exist for children and adolescents aged 6-17 years [12]. For this reason, we developed a test setup based on established test procedures, recommended by the Swiss Federal Institute of Sport [22] and inspired by the “Motorische Basisdiagnostik, MBD” [23], in order to record aspects of health-related fitness as well as motor performance fitness in adult patients with CF.

The present study is aimed at (a) showing longitudinal changes in parameters representing motor performance fitness (balance, coordination, and power), health-related fitness (cardiorespiratory endurance, muscular strength, and flexibility), and HPA during a partially supervised exercise program and (b) investigating whether these parameters are complementary to peak exercise capacity (*W*_peak_), assessed by an incremental cycle test, as additional outcome parameters in exercise programs in adult patients with CF. We assume that the partially supervised exercise program will improve parameters of health-related and motor performance fitness after 6 months as well as lung function, HPA, and BMI. In addition, we suggest that these effects will remain stable after the end of supervision.

## 2. Methods

### 2.1. Subject Selection

The CFmobil project, a 12-month partially supervised training program for patients with CF, was carried out from July 2014 till August 2018 at three regional CF centers (Ruhrlandklinik Essen, University Children's Hospital Essen, and University Children's Hospital Bochum) in Germany. All CF patients ≥ 6 years receiving care at one of the three CF-centers were considered for participation. All patients or their caregivers provided a written informed consent. In the presented analysis, only participants ≥ 18 years were included. The diagnosis of CF was taken from the patient record and was made on the basis of (a) the detection of two CF-defining mutations or (b) two pathological sweat tests (sweat chloride > 60 mmol/L). Exclusion criteria were as follows: Cor pulmonale, decompensated heart failure, musculoskeletal disorders that do not allow continuous training, or an insufficiently treated diabetes mellitus. All relevant clinical patient data were collected from paper or electronically based records. The study was approved by the local ethics committee of the University Hospital Essen (14-6117-BO) and has been registered on ClinicalTrials.gov (NCT03518697).

### 2.2. Study Design

The study was set up as a preexperimental one group pretest-posttest interventional study. Assessment of anthropometry, lung function, physical fitness, and activity was recorded at 3 points in time: baseline (T0), after 6 months (T1), and after 12 months (T2). The subjects participated in an individual training program, which was in collaboration with a sport and exercise scientist. The individual exercise program was developed considering age/gender, body functions, disease-related restrictions, personal factors (individual interests and inclinations), and environmental factors (availability of appropriate training facilities at home, at the place of residence, etc.) [9, 24, 25].

For aerobic training, the main emphasis was to improve aerobic endurance. The training intensity was determined by an incremental cycle ergometer test, and training intensity was set at 70-80% of HR of *W*_peak_. In case of a drop of oxygen saturation < 90% during exercise testing, training intensity was determined on the HR at which the O2 saturation was ≥90%. Duration of training should be 30-40 minutes two to three times/week. If a patient was not able to exercise continuously for 30 to 40 minutes, the time was split up into bouts of 10-15 minutes according to the physical fitness of the patient [9].

Additionally, an individual resistance training program was created with the sport and exercise therapist for each patient including main muscle groups. Depending on the local facilities and personal interests of the patients, the resistance training was performed either with machines (10-15 repetitions, 50% of one-repetition-maximum (1-RM) or a moderate rate of perceived exertion (RPE) 5-6 of a 10-point scale) at a fitness center or at home with Thera-band or tubes of different levels of resistance (10-15 repetitions or a moderate RPE 5-6 of a 10-point scale) which were provided during the project.

According to the Guidance for exercise prescription of the American College of Sports Medicine (ACSM), special attention was paid to flexibility exercise and neuromotor exercise training [24]. All training (intensity of training of strength/endurance, resistance and coordination/balance, and flexibility) recommendations were applied to the patients in written and/or pictured form using the Physiotools software (Physiotools, Tampere, Finland) and were adopted individually according to the patient's individual progress or information.

During the first two months, the participant was contacted by phone twice a week by the sport scientist to identify implementation problems and to address motivational or volitional issues. Subsequently, the participants were contacted by phone every four weeks up to T1 (after six months) or during regular in-/outpatient visits. Problems occurring during exercise were discussed, and appropriate solutions were searched. During the last six months of the intervention (T1 to T2), no contacts were scheduled between the participant and the therapist, except on personal initiative. The training plan could be adapted at any time, if the sport scientist or the treating physicians considered it necessary for sport science, physiotherapy, or medical reasons.

### 2.3. Anthropometry and Lung Function

Forced vital capacity (FVC) and forced expiratory volume in 1 second (FEV1) were measured with a JAEGER MasterScreen Body (CareFusion, Hoechberg, Germany) at all three centers according to ATS guidelines [26]. Body mass index (BMI: kg/m^2^) was calculated using an electronic flat scale (SECA 861, SECA, Hamburg, Germany) and a telescopic measuring rod (SECA 202, SECA, Hamburg, Germany).

### 2.4. Physical Fitness, Incremental Cycle Test, and Habitual Physical Activity (HPA)

The assessment of physical fitness included the following five test items all times: forward bend (FB (cm); flexibility of the hip and trunk), bent knee hip extension (HE (cm); strength/endurance of the lower back), plank leg raise (PLR (sec); pillar stability and strength/endurance), standing long jump (SLJ (cm); power), and standing on one leg (OLS, (sec); coordination/balance).

For test setup, detailed description, and rating, see Table 1. The test composition is based on the recommendations of the Swiss Federal Institute of Sport [22] and the “Motorische Basisdiagnostik, MBD” [23].

Additionally, an incremental cycling test on an electromagnetically braked cycle ergometer (ergoselect 100p, ergoline GmbH, Bitz, Germany) was performed at T0, T1, and T2 according to the Godfrey protocol to measure *W*_peak_ [27]. Heart rate (HR) was continuously recorded using a monitor (polar A300, Polar, Kempele, Finland) and chest strap. Reference equations of Godfrey [27] for *W*_peak_ (%pred.) and Rowland [28] for HR (%pred.) were used. HR_peak_ and *W*_peak_ were determined as the highest value 30 sec before the test was finished.

**Figure 1:**
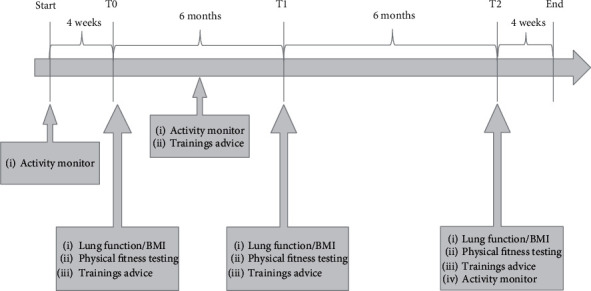
Timeline CFmobil.

HPA (steps/day) was recorded using an activity monitor (wActiSleep-BT Monitor, Actigraph Corp., Pensacola, FL, USA) for a period of 4 weeks each before the intervention has started, after three months, and after 12 months (Figure 1).

### 2.5. Statistical Analysis

Statistical analysis was performed using version 25 of the SPSS statistics package (SPSS Inc., Chicago, USA). Data are presented as mean ± standard deviation (SD). The Shapiro-Wilk test was used to test for normal data distribution. For consecutive physical fitness parameters, results of exercise testing, lung function data, and BMI, a repeated measure analysis of variance (rANOVA) was performed to check for statistical significance. Post hoc analysis was performed by a Bonferroni-adjusted post hoc test. Correlation analysis was done by Pearson correlation. A *p* value ≤0.05 was considered statistically significant.

## 3. Results

### 3.1. Subjects

A total of twenty-six adult patients with CF finished the 12-month exercise program. For distinctive baseline clinical characteristics of the participating subjects, see Table 2.

### 3.2. Health-Related and Motor Performance Fitness

Table 3 shows the results of health-related and motor performance fitness test scores for each test item. All test items showed improvement within the first six months under supervision (FB: *p* ≤ 0.05; PLR and OLS: *p* ≤ 0.01; HE and SLJ: *p* > 0.05). In the further course of the intervention, a small improvement in HE, PLR, and OLS was observed, whereas FB and SLJ showed a slight deterioration. None of these changes showed statistical significance (all *p* > 0.05). Over the entire study period, all five test items showed an overall improvement compared to baseline, with four of the five items improving significantly (FB, PLR, and OLS: *p* ≤ 0.01; HE: *p* ≤ 0.05; SLJ *p* > 0.05).

### 3.3. Bicycle Ergometer Test and Habitual Physical Activity (HPA)

The results of the incremental cycle test (Table 3) showed no statistically significant improvements in *W*_peak_ during the study period (T0: 127 ± 48, T1: 135 ± 52, and T2: 136 ± 51; all *p* > 0.05). Also, we did not find significant changes in HPA (steps/day) during the first months (T0: 8619 ± 2696 and T1: 8902 ± 4127; *p* > 0.05). The numbers of steps/day slightly dropped below the initial level at the end of the intervention (T2: 8560 ± 3560).

### 3.4. Lung Function and BMI

Data collected on lung function and BMI are shown in Table 3. BMI remained stable over the time of intervention (T0: 19.6 ± 2.7, T1: 19.8 ± 3.0, and T2: 19.9 ± 2.8; all *p* > 0.05) whereas a slight deterioration of ppFEV1 (T0: 53.7 ± 21.0, T1: 51.4 ± 22.2, and T2: 51.0 ± 20.6; *p* > 0.05) was recorded.

### 3.5. Correlation between Motor Performance and Bicycle Ergometer Testing

Motor performance fitness test items representing power and strength/endurance (PLR and SLJ) showed a strong correlation to *W*_peak_(PLR: *r*_T0_ = .481, *r*_T1_ = .571, and *r*_T2_ = .530; all *p* ≤ 0.05 and SLJ: *r*_T0_ = .819, *r*_T1_ = .814, and *r*_T2_ = .794; all *p* ≤ 0.01) at each time of measurement (Table 4). In contrast, test items representing balance, coordination, or flexibility (FB, HE, and OLS) showed only a weak (HE: *r*_T0_ = .428; *p* ≤ 0.05) or no correlation to *W*_peak_.

## 4. Discussion

**Figure 2:**
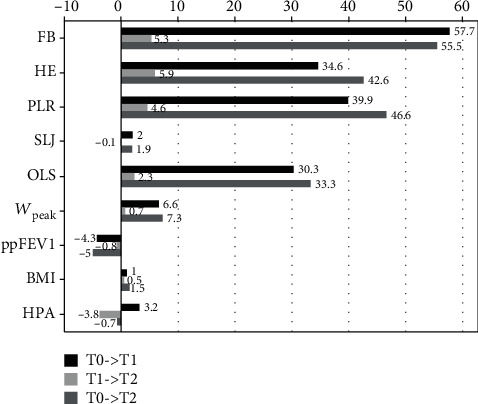
Absolute change (in %) of the individual test items. FB: forward bend; HE: bent knee hip extension; PLR: plank leg raise; SLJ: standing long jump; OLS: standing on one leg; *W*_peak_: peak work load; ppFEV1: percent predicted forced expiratory volume in 1 second; BMI: body mass index; HPA: habitual physical activity.

Our data show the positive influence of a partially supervised exercise training program for adult patients with CF on different aspects of physical fitness (Figure 2). During the supervision, a significant increase in physical fitness (FB, PLR, and OLS) was documented, whereas only a positive trend was shown for HE, SLJ, *W*_peak_, BMI, and HPA. ppFEV1 showed a slight deterioration. After the end of supervision, none of the test items showed a further significant improvement. However, nearly all determined parameters were above baseline (except ppFEV1 and HPA). The assessment of different aspects of PF (balance, coordination, power, muscular strength, flexibility, and cardiorespiratory endurance) was possible by the implementation of an easy-to-perform test setup.

As seen in children with CF [20], our data suggest that there is trainability of adults with CF regarding their physical fitness. Due to a lack of studies, the importance of those parameters in context to CF remains unclear. Studies in children and adolescents with and without CF show a good correlation between items of motor performance fitness and PA. Children with higher levels of motor performance present with a higher level of PA in adolescence and adulthood [29–31]. Transferred to adult patients with CF, it can be speculated that higher levels of motor performance fitness and therefore PA may have a positive impact on the course of the disease, especially concerning to HRQoL and aerobic fitness [32]. Studies outside of CF revealed that deficits in motor performance fitness represent a risk factor for person-related injuries and falls regardless of age and imply that this might be particularly relevant in the context of CF to show their so-called preventive character [33].

In the present study, we could not show significant improvements in ppFEV1 and only a trend towards improvement for exercise capacity (*W*_peak_), as shown in other studies [34–36]. A possible reason for this result may be found in the study design. The intervention did not exclusively focus on aerobic endurance, power, and/or strength but also implied coordinative exercises and resistance training. Exercise capacity is mostly influenced by combining aerobic and anaerobic activities [5] whereas lung function is commonly affected by peripheral muscle strength [37]. This may explain why no training effect could be detected on *W*_peak_ and ppFEV1. Our results are more in line with the results reported by Schneiderman-Walker al. [38] and Hebestreit et al. [13] who did not find a positive effect on ppFEV1 in their exercise programs either. In addition, it remains unclear whether all participants adhered to the instructions of the sport therapist.

Undoubtedly, the implementation of CPET to determine VO2_max_ and VO2_peak_ plays an important role in CF patient care. The ECFS Exercise Working Group recommends CPET to evaluate aerobic exercise capacity and the effects of a training intervention [19]. A study by Hebestreit et al. [18] showed the high prognostic value of those parameters in CF. This is why CPET-derived parameters, besides pulmonary function (mostly expressed as ppFEV1) and health-related quality of life (HRQoL), are often regarded as the main outcome parameters in studies determining the influence of exercise programs on performance in patients with CF. Those parameters reflect a major part of health-related fitness but cannot be used to make a statement about motor performance fitness level of the individual. Like in our study, where CPET was not always feasible, the measurement of *W*_peak_ can be done by an incremental cycle test according to the ECFS guidelines [19]. *W*_peak_ correlated well to markers of (explosive) power (SLJ) and endurance/strength (PLR). OLS, HE, and FB showed no correlation in this regard. This allows the conclusion that a statement about exercise performance can be made by means of a simple fitness test.

However, study designs reported in literature vary greatly with regard to types of intervention (anaerobic, aerobic, combined aerobic and anaerobic training, strength training, supervised, partially supervised, and unsupervised), duration of the programs, and characteristics of the participants [5]. Thus, comparability of the reported results is limited.

Compared to formerly described test setups for adult patients without CF [21, 39], the implementation of our test setup into clinical routine was equally easy, because time and staff requirements are low, the test procedure was easy to understand, and the equipment and space requirements were low. During the entire intervention phase, no side effects were observed among the participants as described in previous trials dealing with sport interventions resembling a good overall safety profile [40].

A Cochrane review performed in 2013 described a lack of studies that prove the positive effect of (partially) supervised training on participation in exercise programs in CF [41]. We do assume that close personal contact and individualized counselling are major factors to promote participation in CF training programs. Our study demonstrates the positive influence of close supervision by experienced sport therapists not only on the improvement in health-related and motor performance fitness but also on recorded HPA. Once the personal counselling of the participants was reduced, only a small further increase or, in some aspects, decrease was detectable. However, the participants remained stable within the last months at the level previously achieved. This assumption is supported by the work of Hommerding et al. [42]. Sport therapists offering assistance at any time to address specific sport questions seem to have a positive impact on the adherence to exercise programs. In addition, our data are consistent with other studies in which a long-lasting effect on participants was observed even after the end of the intervention [13, 34]. Many known barriers are known in patients with CF preventing them from regular PA [43]. To counteract this at an early stage, patient education and support are of enormous importance. Here, too, the continuous care of the individual by trained staff in promoting PA can be helpful to dispel fears and worries [44].

The present study has several limitations. A major drawback is the absence of a nontraining control group which makes it difficult to analyze the effects of the exercise program on the participants. The other main limitation is the lack of comparability with the gold standard CPET. A statement on comparability between improvements in PF, seen in our study, and CPET parameters VO2_peak_ and VO2_max_ was not possible in our test setting and would be of great interest. In addition, the selected test items to assess physical fitness are not validated for adults with CF but our data show that the chosen setup can serve as a basis for the development of a new test.

## 5. Conclusions

A 12-month partially supervised exercise program shows the trainability of and positive effects on physical fitness in adult patients with CF. With the help of a simple test setup, these effects can be tested in relation to power, strength/endurance, balance/coordination, and flexibility. The most distinct improvements can be achieved under close supervision by an experienced sport therapist. Besides the established parameters, expanded testing of motor performance and health-related fitness can provide valuable additional information about the impact of activity and exercise programs on adult patients with CF. Further clinical studies are needed to validate an enhanced physical fitness test in order to include these parameters in future clinical trials as possible additional outcome parameters.

## Figures and Tables

**Table 1:** Physical fitness test items and setup.

	Forward bend (FB)	Bent knee hip extension (HE)	Plank leg raise (PLR)	Standing long jump (SLJ)	Standing on one leg (OLS)
Goals	Detection of mobility in the hip and lumbar spine area	Function and performance assessment of the gluteal muscles	Strength of the global core muscles	Power of the legs	Coordinative skills
Test setup	The test procedure requires a 20 to 30 cm high chair with a centimeter scale.	The test is performed while lying on a gymnastics mat.	The test is performed on a gymnastics mat.	A measuring tape/marking is fixed on the floor perpendicular to show the jump line. The jump line is to be marked. The zero point of the measuring tape is at the front edge of the jump line.	Subject is standing in a tire.
Test description	The subject stands on a chair and bends forward with his knees bent. With the arms stretched down, an attempt is made to reach the lowest possible measuring point and to hold for 2 seconds. The finger-floor distance is measured in centimeter. The zero point of the scale is the floor level. Positive values are readings lower than the sole level; negative values mean that the sole level has not been reached. In total, two measurements were performed.	The subject is lying in prone position. The knees are bent. The hands are crossed on the back. The subject attempts to lift the knees off the ground for 5 seconds. The head remains on the floor during the exercise.	In the specified position (forearm support), the left and right feet are raised off the ground by half a foot each second. The time is measured until the abortion. The measurement is aborted when the subject returns to the prone position, turns off the knees, or rests his head on the arms.	The subject must jump off the stand with both legs at the same time; both feet are in front of the jump line and must touch the ground until they jump off. Failed attempts are invalid. Before the jump, the candidates should easily go to their knees, achieve a good preload, take light pattern, and jump off sharply. Rockers are allowed, do not hurry.	After 10 seconds, the eyes must be closed. After another 10 seconds, the head must be placed backwards with the eyes closed. The time to loss of balance is measured for the left and right legs.
Rating	The best value of the two runs is scored.	The exercise is solved when the tension is held for 5 seconds and no back pain occurs.	The time is measured to a second exactly.	Measurements are made to the nearest centimeter, from the leading edge of the drop line to the rearmost point of contact of the body on the ground (usually the heel).	One-legged left leg time sums up one-legged right leg time. After 60 seconds, the measurement is aborted and the maximum time of 60 seconds is entered.

**Table 2:** Patient characteristics and pulmonary function data.

	Participants (*n* = 26)
Age, years	26.5 ± 7.9 (19–50)
Gender	
Female, *n* (%)	8 (31)
Male, *n* (%)	18 (69)
Genotype	
F508del homozygous, *n* (%)	17 (65)
F508del heterozygous, *n* (%)	6 (23)
Other, *n* (%)	3 (12)
BMI, kg/m^2^	19.6 ± 2.7 (16-28)
FEV1, L	2.1 ± 1.0 (0.7–4.4)
FEV1, % predicted	53.7 ± 21.0 (17–98)
FVC, L	3.2 ± 1.2 (1.6–5.7)
FVC, % predicted	67.7 ± 20.0 (28–102)
Pancreatic insufficiency, *n* (%)	21 (81)
*P*. *aeruginosa* positive, *n* (%)	15 (58)
Cystic fibrosis-related diabetes, *n* (%)	7 (27)
O2 supplementation, *n* (%)	4 (15)

Results are presented as mean ± standard deviation (SD) and range or number of patients *n* (%). BMI: body mass index; FEV1: forced expiratory volume in 1 second; FVC: forced vital capacity.

**Table 3:** Results of physical fitness and bicycle ergometer testing, lung function (FEV1), and BMI at baseline (T0), after six (T1), and after 12 months (T2).

	T0 (baseline)	T1 (after 6 months)	T2 (after 12 months)	rANOVA	Post hoc analysis		
				*T*	T0->T1	T1->T2	T0->T2
FB—forward bend (cm) (*n* = 24)	−4.5 ± 13.5	−1.9 ± 12.1	−2.0 ± 10.9	*p* ≤ 0.01	*p* ≤ 0.05	*p* > 0.05	*p* ≤ 0.05
HE—bent knee hip extension (sec) (*n* = 21)	39.0 ± 17.7	52.5 ± 39.8	55.6 ± 41.2	*p* ≤ 0.05	*p* > 0.05	*p* > 0.05	*p* > 0.05
PLR—plank leg raise (sec) (*n* = 18)	56.4 ± 27.0	78.9 ± 49.3	82.7 ± 41.3	*p* ≤ 0.01	*p* ≤ 0.01	*p* > 0.05	*p* ≤ 0.01
SLJ—standing long jump (cm) (*n* = 19)	156.3 ± 35.7	159.4 ± 37.1	159.2 ± 36.3	*p* > 0.05	*p* > 0.05	*p* > 0.05	*p* > 0.05
OLS—standing on one leg (sec) (*n* = 24)	49.5 ± 14.3	64.5 ± 33.2	66.0 ± 38.0	*p* ≤ 0.01	*p* ≤ 0.01	*p* > 0.05	*p* ≤ 0.05
*W*_peak_ (W) (*n* = 22)	127.1 ± 48.7	135.5 ± 52.1	136.4 ± 51.2	*p* > 0.05	*p* > 0.05	*p* > 0.05	*p* > 0.05
*W*_peak_ (%pred.) (*n* = 22)	59.1 ± 19.8	63.5 ± 20.7	64.0 ± 18.3	*p* > 0.05	*p* > 0.05	*p* > 0.05	*p* > 0.05
*W*_peak/kg_ (*n* = 22)	2.1 ± 0.7	2.2 ± 0.7	2.2 ± 0.6	*p* > 0.05	*p* > 0.05	*p* > 0.05	*p* > 0.05
HR/min rest (*n* = 22)	99.1 ± 15.8	101.6 ± 15.1	97.7 ± 14.7	*p* > 0.05	*p* > 0.05	*p* > 0.05	*p* > 0.05
HR/min max (*n* = 22)	159.2 ± 21.9	163.4 ± 21.0	161.1 ± 22.4	*p* > 0.05	*p* > 0.05	*p* > 0.05	*p* > 0.05
FEV1 (%pred.) (*n* = 26)	53.7 ± 21.0	51.4 ± 22.2	51.0 ± 20.6	*p* > 0.05	*p* > 0.05	*p* > 0.05	*p* > 0.05
BMI (kg/m^2^) (*n* = 26)	19.6 ± 2.7	19.8 ± 3.0	19.9 ± 2.8	*p* > 0.05	*p* > 0.05	*p* > 0.05	*p* > 0.05
Steps/day (*n* = 17)	8619 ± 2696	8902 ± 4127	8560 ± 3560	*p* > 0.05	*p* > 0.05	*p* > 0.05	*p* > 0.05

Results are presented as mean ± standard deviation (SD). *n*: number of patients with complete data sets. *W*_peak_: peak work load (watts); FEV1: forced expiratory volume in 1 second; BMI: body mass index; *T*: time; rANOVA: repeated measure analysis of variance.

**Table 4:** Pearson's correlation between results of physical fitness and bicycle ergometer testing at baseline (T0), after six (T1), and after 12 months (T2).

	*W*_peak_ T0	*W*_peak_ T1	*W*_peak_ T2
FB	*r* = −.179; *p* > 0.05	*r* = −.161; *p* > 0.05	*r* = −.265; *p* > 0.05
HE	*r* = .428; *p* ≤ 0.05	*r* = .413; *p* > 0.05	*r* = .259; *p* > 0.05
PLR	*r* = .481; *p* ≤ 0.05	*r* = .571; *p* ≤ 0.05	*r* = .530; *p* ≤ 0.05
SLJ	*r* = .819; *p* ≤ 0.01	*r* = .814; *p* ≤ 0.01	*r* = .794; *p* ≤ 0.01
OLS	*r* = −.031; *p* > 0.05	*r* = −.133; *p* > 0.05	*r* = −.391; *p* > 0.05

FB: forward bend (cm); HE: bent knee hip extension (sec); PLR: plank leg raise (sec); SLJ: standing long jump (cm); OLS: standing on one leg (sec); *W*_peak_: peak work load (watts); *r*: Pearson's correlation coefficient.

## Data Availability

The datasets used and/or analyzed during the current study are available from the corresponding author on reasonable request.

## Abbreviations

BMI: Body mass index
CF: Cystic fibrosis
CFRD: Cystic fibrosis-related diabetes
CPET: Cardiopulmonary exercise testing
FB: Forward bend
FEV1: Forced expiratory volume in one/1 second
FVC: Forced vital capacity
HE: Hip extension
HPA: Habitual physical activity
HRQoL: Health-related quality of life
OLS: One leg stand
PLR: Plank leg raise
RM: Repetition maximum
RPE: Rate of perceived exertion
SLJ: Standing long jump
VO2_max_: Maximum oxygen uptake
VO2_peak_: Peak oxygen uptake
W: Watts
*W*_peak_: Peak workload.

## References

[1] Mehta G., Macek M., Mehta A. (2010). Cystic fibrosis across Europe: EuroCare CF analysis of demographic data from 35 countries. *Journal of Cystic Fibrosis*.

[2] Bell S. C., Mall M. A., Gutierrez H. (2020). The future of cystic fibrosis care: a global perspective. *The Lancet Respiratory Medicine*.

[3] Kerem E., Reisman J., Corey M., Canny G. J., Levison H. (1992). Prediction of mortality in patients with cystic fibrosis. *The New England Journal of Medicine*.

[4] Stephenson A. L., Stanojevic S., Sykes J., Burgel P.-R. (2017). The changing epidemiology and demography of cystic fibrosis. *Presse Médicale*.

[5] Radtke T., Nevitt S. J., Hebestreit H., Kriemler S. (2017). Physical exercise training for cystic fibrosis. *Cochrane Database of Systematic Reviews*.

[6] Williams C. A., Stevens D. (2013). Physical activity and exercise training in young people with cystic fibrosis: current recommendations and evidence. *Journal of Sport and Health Science*.

[7] Howley E. T. (2001). Type of activity: resistance, aerobic and leisure versus occupational physical activity. *Medicine and Science in Sports and Exercise*.

[8] Caspersen C. J., Powell K. E., Christenson G. M. (1985). Physical activity, exercise, and physical fitness: definitions and distinctions for health-related research. *Public Health Reports*.

[9] Swisher A. K., Hebestreit H., Mejia-Downs A. (2015). Exercise and habitual physical activity for people with cystic fibrosis. *Cardiopulmonary Physical Therapy Journal*.

[10] Haga M. (2008). The relationship between physical fitness and motor competence in children. *Child: Care, Health and Development*.

[11] Gísladóttir O., Haga M., Sigmundsson H. (2014). Motor competence and physical fitness in adolescents. *Pediatric Physical Therapy*.

[12] Bös K., Schlenker L. (2009). *Deutscher Motorik-Test 6-18: (DMT 6-18)*.

[13] Hebestreit H., Kieser S., Junge S. (2010). Long-term effects of a partially supervised conditioning programme in cystic fibrosis. *The European Respiratory Journal*.

[14] Pianosi P., LeBlanc J., Almudevar A. (2005). Peak oxygen uptake and mortality in children with cystic fibrosis. *Thorax*.

[15] Schneiderman J. E., Wilkes D. L., Atenafu E. G. (2014). Longitudinal relationship between physical activity and lung health in patients with cystic fibrosis. *The European Respiratory Journal*.

[16] Pianosi P., LeBlanc J., Almudevar A. (2005). Relationship between FEV1 and peak oxygen uptake in children with cystic fibrosis. *Pediatric Pulmonology*.

[17] Vendrusculo F. M., Heinzmann-Filho J. P., da Silva J. S., Perez Ruiz M., Donadio M. V. F. (2019). Peak oxygen uptake and mortality in cystic fibrosis: systematic review and meta-analysis. *Respiratory Care*.

[18] Hebestreit H., Hulzebos E. H. J., Schneiderman J. E. (2019). Cardiopulmonary exercise testing provides additional prognostic information in cystic fibrosis. *American Journal of Respiratory and Critical Care Medicine*.

[19] Hebestreit H., Arets H. G. M., Aurora P. (2015). Statement on exercise testing in cystic fibrosis. *Respiration*.

[20] Gruber W., Stehling F., Olivier M. (2020). Effects of a long-term exercise program on motor performance in children and adolescents with CF. *Pediatric Pulmonology*.

[21] Rikli R. E., Jones C. J. (1999). Development and validation of a functional fitness test for community-residing older adults. *Journal of Aging and Physical Activity*.

[22] (2016). *Eidgenössische Hochschule für Sport Magglingen EHSM. Sport in der Armee: Fitnesstest der Armee FTA für die Rekrutierung*.

[23] Wydra G., Schüle K., Huber G. (2000). Problemorientierte Diagnosestrategie in der Sporttherapie. *Grundlagen der Sporttherapie*.

[24] Garber C. E., Blissmer B., Deschenes M. R. (2011). American College of Sports Medicine position stand. Quantity and quality of exercise for developing and maintaining cardiorespiratory, musculoskeletal, and neuromotor fitness in apparently healthy adults: guidance for prescribing exercise. *Medicine and Science in Sports and Exercise*.

[25] Thornton J. S., Frémont P., Khan K. (2016). Physical activity prescription: a critical opportunity to address a modifiable risk factor for the prevention and management of chronic disease: a position statement by the Canadian Academy of Sport and Exercise Medicine. *British Journal of Sports Medicine*.

[26] Miller M. R., Hankinson J., Brusasco V. (2005). Standardisation of spirometry. *European Respiratory Journal*.

[27] Godfrey S., Mearns M. (1971). Pulmonary function and response to exercise in cystic fibrosis. *Archives of Disease in Childhood*.

[28] Rowland T. W. (1996). *Developmental Exercise Physiology*.

[29] Cattuzzo M. T., dos Santos Henrique R., Ré A. H. N. (2016). Motor competence and health related physical fitness in youth: a systematic review. *Journal of Science and Medicine in Sport*.

[30] Gruber W., Braumann K. M., Orenstein D. M., Hüls G. (2006). Effects of an exercise program on motor performance in CF-Patients. *Pediatric Pulmonology*.

[31] Stodden D. F., Goodway J. D., Langendorfer S. J. (2008). A developmental perspective on the role of motor skill competence in physical activity: an emergent relationship. *Quest*.

[32] Hebestreit H., Schmid K., Kieser S. (2014). Quality of life is associated with physical activity and fitness in cystic fibrosis. *BMC Pulmonary Medicine*.

[33] Muehlbauer T., Gollhofer A., Granacher U. (2015). Associations between measures of balance and lower-extremity muscle strength/power in healthy individuals across the lifespan: a systematic review and meta-analysis. *Sports Medicine*.

[34] Kriemler S., Kieser S., Junge S. (2013). Effect of supervised training on FEV_1_ in cystic fibrosis: a randomised controlled trial. *Journal of Cystic Fibrosis*.

[35] Selvadurai H. C., Blimkie C. J., Meyers N., Mellis C. M., Cooper P. J., van Asperen P. P. (2002). Randomized controlled study of in-hospital exercise training programs in children with cystic fibrosis. *Pediatric Pulmonology*.

[36] Santana Sosa E., Groeneveld I. F., Gonzalez-Saiz L. (2012). Intrahospital weight and aerobic training in children with cystic fibrosis: a randomized controlled trial. *Medicine and Science in Sports and Exercise*.

[37] Rovedder P. M. E., Borba G. C., Anderle M. (2019). Peripheral muscle strength is associated with lung function and functional capacity in patients with cystic fibrosis. *Physiother Res Int*.

[38] Schneiderman-Walker J., Pollock S. L., Corey M. (2000). A randomized controlled trial of a 3-year home exercise program in cystic fibrosis. *The Journal of Pediatrics*.

[39] Cha J.-Y., Min S.-K., Yoon T.-H., Jee Y.-S. (2020). Gross motor function and health fitness in adults with autistic spectrum disorder and intellectual disability: single-blind retrospective trial. *Journal of Exercise Rehabilitation*.

[40] Ruf K., Winkler B., Hebestreit A., Gruber W., Hebestreit H. (2010). Risks associated with exercise testing and sports participation in cystic fibrosis. *Journal of Cystic Fibrosis*.

[41] Cox N. S., Alison J. A., Holland A. E. (2013). Interventions for promoting physical activity in people with cystic fibrosis. *Cochrane Database of Systematic Reviews*.

[42] Hommerding P. X., Baptista R. R., Makarewicz G. T. (2015). Effects of an educational intervention of physical activity for children and adolescents with cystic fibrosis: a randomized controlled trial. *Respiratory Care*.

[43] Denford S., van Beurden S., O'Halloran P., Williams C. A. (2020). Barriers and facilitators to physical activity among children, adolescents, and young adults with cystic fibrosis: a systematic review and thematic synthesis of qualitative research. *BMJ Open*.

[44] Denford S., Mackintosh K. A., McNarry M. A., Barker A. R., Williams C. A. (2019). Enhancing intrinsic motivation for physical activity among adolescents with cystic fibrosis: a qualitative study of the views of healthcare professionals. *BMJ Open*.

